# Management of a Unique Presentation of a Common Dermatologic Condition

**DOI:** 10.51894/001c.24501

**Published:** 2021-08-30

**Authors:** Casey P. Schukow, Madeline Schaeffer, Katherine Boss, David Fivenson

**Affiliations:** 1 Michigan State University College of Osteopathic Medicine; 2 Dermatology St. Joseph Mercy Health

**Keywords:** macrophage, corticosteroid, steroid, biopsy, patch granuloma annulare, granuloma annulare

## Abstract

**CONTEXT:**

Skin rashes are a common complaint seen in the primary care setting. There are many dermatologic conditions which a primary care provider (PCP) should be able to recognize and manage. One such condition is granuloma annulare (GA), which commonly presents as smooth, annular plaques on the trunk and/or extremities. Rashes like GA rarely present as unique variants and may be difficult for PCPs to determine from patient history and physical exam alone. Patch granuloma annulare (patch GA) is an example that may clinically mimic a cutaneous lymphoma known as mycosis fungoides (MF). PCPs should ideally be able to recognize the utility of performing a skin biopsy and/or referring the patient to a dermatologist when history and physical exam alone are insufficient. The histologic findings of skin biopsies often become essential in establishing a proper diagnosis and guiding patient management in unique dermatologic variants.

**EXAMPLE CASE:**

The patient in this clinical practice report is a Caucasian female in her late 60s who presented to a dermatology clinic with a two-year history of a worsening widespread eruption on her trunk and extremities. She had been evaluated previously by her PCP about 4 months prior and, without obtaining skin biopsies, treated her with a medium potency topical corticosteroid cream. The eruption had spread over her hips, buttocks, back, thighs, wrists, and elbows. Multiple skin biopsies of affected sites were taken by the second author and revealed findings consistent with patch GA. The patient was started on topical betamethasone dipropionate 0.05% ointment twice daily and noted marked improvement of her symptoms.

**CONCLUSIONS:**

Although GA is a benign condition of the skin that may be readily detected by PCPs, skin biopsies may be necessary to establish a proper diagnosis when this condition presents as a unique variant (e.g., patch GA). Therapy for patch GA often begins with a trial of high-potency topical steroid therapy in combination with ultraviolet light exposure, depending on disease severity and patient preference. Early evaluation with a skin biopsy by her PCP or an earlier referral to a dermatologist to have skin biopsies performed likely would have helped establish a prompter diagnosis and treatment plan for this patient.

## INTRODUCTION

A study conducted by Lowell et. al. in 2001 determined that approximately one in three patients present to their primary care provider (PCP) with at least one dermatologic complaint (e.g., “rash”).^1^ In this same study, the authors also concluded that roughly 37.5% of patients presenting with rashes are referred from their PCP to a dermatologist.^1^ Lowell et. al. also noted that PCPs made the correct diagnosis of rashes when compared to dermatologist evaluation approximately 57% of the time.^1^

With the average age of persons in the United States (U.S.) continuing to increase, a relative shortage of dermatologists remain.^2^ Thus, more patients are being managed by PCPs for their dermatologic conditions as the U.S. population grows.^3^ While online modules and practice during residency training do help give PCPs a fundamental background behind many common dermatologic conditions (e.g., acne, seborrheic keratosis), most PCPs still have difficulty managing these patients, especially when their presentations are atypical.^3,4^ This issue among PCP training continues to be addressed through practice resources and dermatologist-established recommendation toolkits.^4,5^

**Figure 1.**
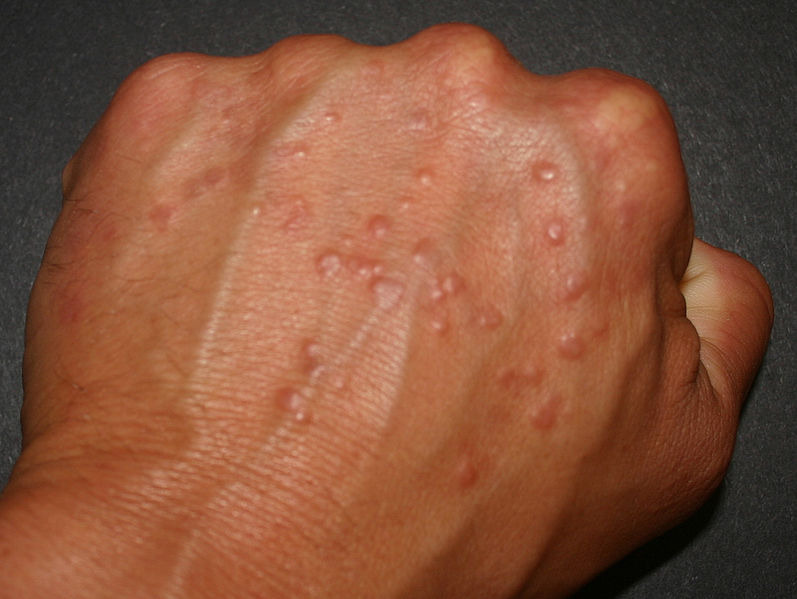
An example of GA on the dorsum of the hand presenting as multiple, red papules. Retrieved via public domain Wikipedia.^7^

One condition that tends to be recognized by PCPs is granuloma annulare (GA), which involves a benign accumulation of macrophages called a “granuloma” in the skin’s dermis.^6^ This condition typically occurs in children under the age of 10 and adults over the age of 40, and affects women twice as often as men.^6^ GA often presents as elevated, small (i.e., “papular”) and/or large (i.e., “plaque”) red lesions in ring-like (i.e., “annular”) formations on the trunk and/or extremities (Figure 1).^6^

Patch GA is a unique clinical variant of GA, with smooth erythematous-to-brown patches on the trunk and/or extremities, as opposed to red papules or annular plaques.^6,8^ It is diagnosed most frequently in females over age 50.^6^ While the annual incidence and prevalence of GA is approximately 0.1% to 0.4%, the number of cases of patch GA presenting to PCPs and even dermatologists are far less.^6,9^ For example, an institution-based, 10-year review of a dermatopathology database (2009-2019) determined 23 out of 108 (21.3%) potential histologic cases resembling GA to be specifically of the patch variant.^10^

Different types of biopsies can be performed on skin lesions by PCPs who feel comfortable doing so after adequate training.^11^ Shave biopsies tend to remove protruding portions of skin lesions, while excisional biopsies remove lesions in their entirety.^11^ Biopsies most appropriate for observing the full skin thickness of a lesion are punch biopsies.^7,11^ These can adequately sample the various layers of the skin (i.e., epidermis, dermis, and subcutaneous fat), giving pathologists enough tissue for proper histologic evaluation.^11,12^ All biopsies should be taken with relative caution as they may inadequately heal if taken with improper technique or if the biopsy site is not properly cared for.^11,12^

Patch GA may also appear similarly to a cutaneous lymphoma known as mycosis fungoides (MF), thus a skin biopsy becomes a vital step in proper diagnosis.^13,14^ Often used to evaluate tissue samples, hematoxylin and eosin (H&E) staining reveals unusual, or “atypical”, lymphocytes favoring the epidermis over the dermis (i.e., “epidermotropism”) in MF.^13,14^ This is different from patch GA, which shows macrophages (“histiocytes”) accumulating in the dermis, scattered around degrading collagen and blood vessels in an “interstitial” pattern, and/or surrounding decaying (“necrobiotic”) tissue in a “palisading” pattern.^7,10,15^

The treatment of patch GA is often multifactorial.^16^ A 2015 systematic review recommended using topical corticosteroids with or without ultraviolet (UV) phototherapy as an initial treatment plan.^16^ This is opposed to MF, which may be treated with topical corticosteroids, but often requires alternative therapy and/or a more prolonged treatment course.^17^ More importantly, patients with advanced MF require a systemic evaluation, often with hematologic analysis and radiologic imaging, to rule-out systemic disease.^17^ Therefore it is imperative that the correct diagnosis be made initially so an underlying malignancy is not missed. In this clinical practice report, the management of a patient who presented with patch GA is followed. Further, the roles of skin biopsies, H&E evaluation, and recognizing patient circumstances by providers (i.e., during a global pandemic) will be discussed.

## EXAMPLE PATIENT DESCRIPTION

A Caucasian woman in her late 60s with no pertinent past medical history presented to the dermatology clinic with a two-year history of a worsening widespread eruption on her trunk and extremities. The eruption was initially localized to her hips and posterior thighs. Over several months, it had spread to also involve her buttocks, upper and lower back, anterior thighs, wrists, and elbows.

The patient stated that the rash was mildly itchy and sometimes burned. She had been seen by her PCP approximately four months prior for this rash and was prescribed triamcinolone 0.1% cream, which she had admitted to using intermittently with some improvement of symptoms. No skin biopsies were obtained at that prior visit. After she stopped using the triamcinolone cream, she stated the eruption worsened. She reported feeling very emotionally stressed lately, as she was the primary caregiver for her husband who was recently transitioned to home hospice.

**Figure 2.**
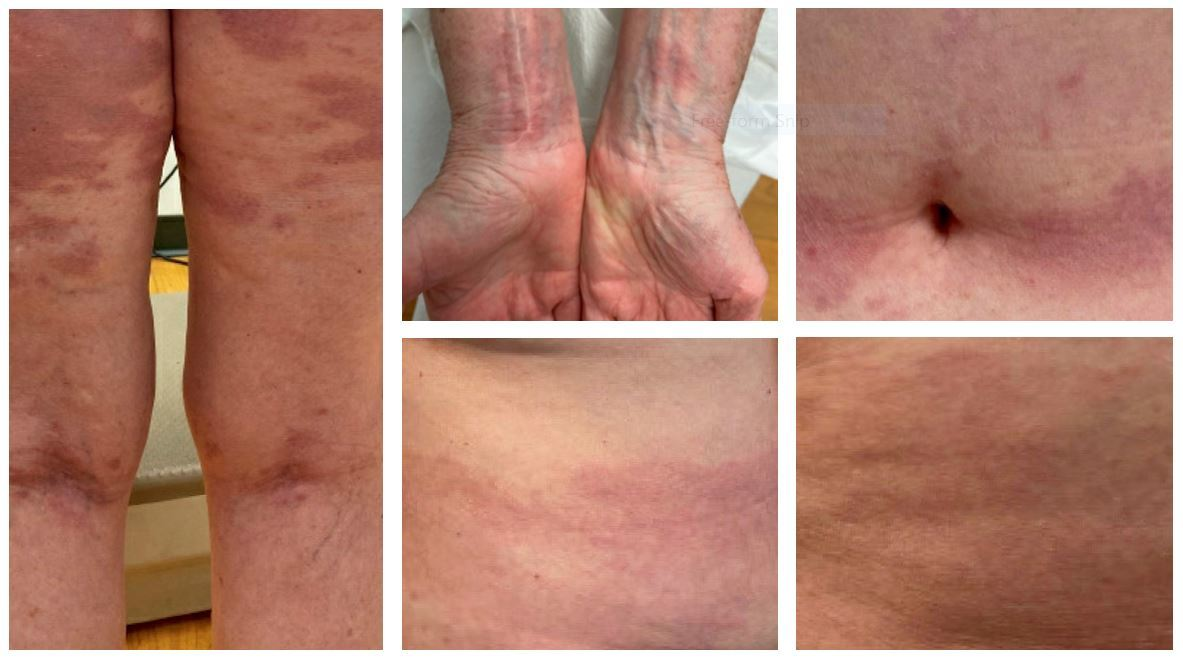
Photographic images of red-brown patches on the bilateral posterior thighs (Left), anterior abdominal skin folds near the umbilicus bilaterally (upper Right), bilateral anterior wrists (upper Middle), and bilateral flanks (lower Middle/Right). Images were cropped and modified using the “Snipping Tool” feature on Microsoft. These photographs were taken after the patient signed a written consent form releasing her photos for educational purposes and publication in the Spartan Medical Research Journal.

On physical exam, several red-brown patches were present on the bilateral flanks, buttocks, posterior thighs, anterior thighs, abdominal skin folds, and the back of her knees (i.e., “popliteal fossae”). There were scattered pink papules on her posterior shoulders, elbows, and ventral wrists, too. On her wrists, some of the papules coalesced to form plaques (see Figure 2).

**Figure 3.**
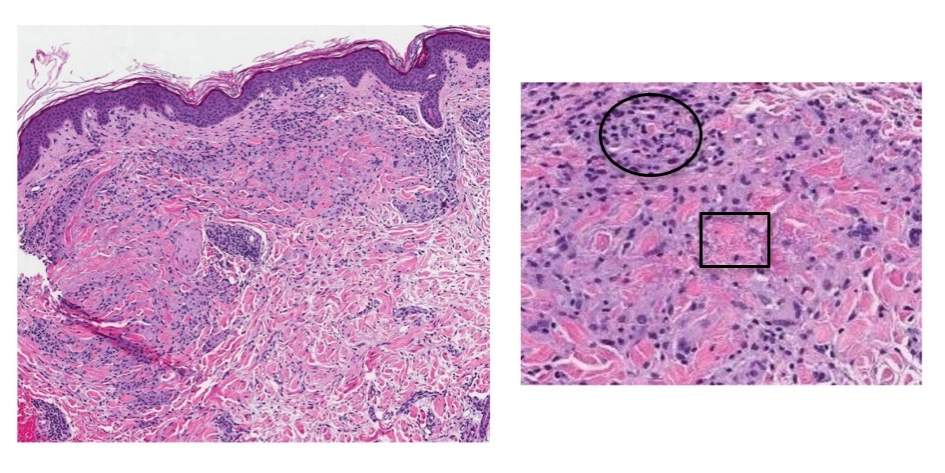
(Left) Histopathologic examination with H&E staining at 10x magnification of one of the punch biopsies taken. (Right) Higher power analysis shows central necrobiosis and mucin deposition.^6,8^ The light purple cells in both images are histiocytes (circle in Right) and they are coalescing around pinkish-blue degraded collagen (square in Right).^6,8^ Images were cropped and modified using the “Snipping Tool” feature on Microsoft.

Contemplating the possible etiologies of this rash, the second author (MS) of this report obtained punch biopsies from a papule on her left posterior shoulder, a patch on her left flank, and a patch on her left thigh. All biopsies showed similar findings: a dense infiltrate of histiocytes forming palisading granulomas within the upper dermis. The epidermis did not show any atypical lymphocytes. Necrobiosis of connective tissue material was also appreciated (see Figure 3).

These histopathologic findings, in accordance with the patient’s history and physical exam findings, confirmed a diagnosis of patch GA. The patient was prescribed topical betamethasone dipropionate 0.05% ointment twice daily, which is a more potent steroid than her previously used triamcinolone 0.1% cream. Weekly UV phototherapy was also recommended. However, the patient elected to proceed with topical therapy only as it would be too challenging to present for regular UV treatments give her home situation.

Unfortunately, the patient has not yet been able to come back into the clinic for in-person follow-up. However, she was contacted on the phone by the second author (MS) of this report several weeks after her initial visit and was able to provide information on the status of her condition. She reported that the topical betamethasone almost entirely cleared her eruption. As she was the main care provider for her husband on home hospice, she had been unable to leave her home much. The COVID-19 pandemic concerned her, too, as she feared leaving the house and contracting the COVID-19 virus. Overall, she was very pleased with the results of the treatment and expressed gratitude towards the care that was provided.

## DISCUSSION

One other 2015 report presented a patient with patch GA confused for MF.^18^ In that report, the authors also acknowledged how taking skin biopsies was essential in managing their patient.^18^ Patch GA is often a self-limiting conditioning and does not impact a patient’s life expectancy.^6,8^ This contrasts with MF, which leaves patients with less than 1.5 years to greater than 11 years life expectancy remaining depending on disease stage.^17^

Skin biopsies have been established as an essential way to diagnose many skin conditions, both malignant and non-malignant.^19,20^ As with any other chief complaint, however, PCPs should first utilize good history taking and physical exam skills (e.g., palpation) to establish a differential diagnosis.^21^ A 2014 study demonstrated how PCPs can play an essential role in ruling out severe skin conditions (e.g., cancer) in regions where dermatologist evaluations are limited.^22^

Although skin biopsies aid in diagnosis, a 2020 report demonstrated that non-dermatology physicians (e.g., PCPs) take an average of 4.55 biopsies to diagnose one skin cancer, versus 2.82 for dermatologists.^23^ This demonstrates a potential over-reliance on skin biopsies in some primary care settings as most rashes presenting to PCPs are non-life threatening.^24^

Skin biopsies should be utilized when history and physical exam is insufficient in providing an accurate diagnosis of clinical variants and/or to help rule out severe conditions such as cancer.^25^ Although there are multiple different types of skin biopsies, a punch biopsy was considered the best choice for the patient in this report, as it allowed for full skin-thickness evaluation.^25^ One biopsy site may be sufficient for localized, solitary lesions.^25^ Widespread lesions, as presented in this patient, necessitated multiple punch biopsies from different locations to ensure the rash was the same throughout.^25^

H&E staining is widely used when analyzing biopsy samples; it provides visualization of the various layers of the skin, its cellular components, and the tissue surrounding each cell (i.e., “extracellular matrix”).^26,27^ Hematoxylin is a basic, or cationic, dye that binds to negatively charged molecular components.^26,27^ Some examples include genetic material within a cell’s nucleus and ribosomes in a cell’s rough endoplasmic reticulum (RER).^26,27^ This dye appears “blue” on H&E and represents basophilic staining.^26,27^

**Figure 4.**
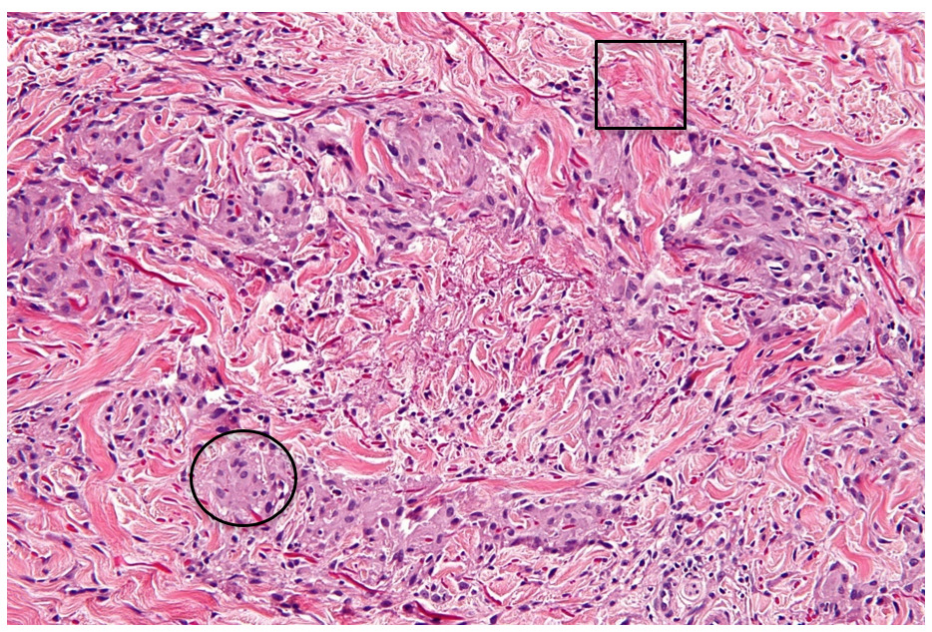
Example H&E of granuloma annulare. Basophilic components (example circled) often represent genetic material, such as DNA within a cell’s nucleus.^26,27^ Eosinophilic components (example squared) often represent proteins, such as intracellular organelles or the collagen fibrils within the extracellular matrix.^26,27^ Image retrieved via public domain Wikipedia.^7^

Eosin, on the other hand, is an acidic, or anionic, dye and binds to positively charged molecular components.^26,27^ Examples most often include positively charged amino acids on intra- and extra-cellular proteins, such as organelles (intracellular) and collagen (extracellular).^26,27^ This dye appears “pink” on H&E and represents eosinophilic staining.^26,27^ Substances which are relatively neutral in charge, then, appear clearer on H&E, as in the case of extracellular mucin.^26,27^ Figure 4 shows an example of a granuloma annulare H&E stain to show these concepts.

This patient was originally prescribed triamcinolone 0.1% cream, which she said she applied once daily for four months. However, she had minimal symptom improvement, so she was prescribed topical betamethasone dipropionate 0.05% ointment to be applied twice daily instead. After several weeks, she had begun seeing resolution of her symptoms. Betamethasone dipropionate 0.05% ointment is stronger than triamcinolone 0.1% cream because the molecule itself (betamethasone versus triamcinolone) is more potent.^28,29^

UV phototherapy is also a treatment option for patients with patch GA and was offered to this patient in addition to topical corticosteroids.^30^ This therapy is not preferred over steroidal interventions due to frequent in-patient visits over the course of many weeks; in some patients, though, it offers complete remission of widespread GA.^31^

As mentioned earlier, the patient in this report was the primary caregiver of her husband in home hospice during the time of her evaluation. Not only did this impact her in making frequent outpatient follow-up visits, but the status of the COVID-19 pandemic left her fearful of becoming ill and passing it on to her husband.^32^ Providing empathetic care and working alongside patients to determine best management plans for their dermatologic condition exemplifies osteopathic principles and philosophy.^33^

## CONCLUSIONS

Many PCPs see patients with a dermatologic complaint, especially in regions where dermatologists are few. While proper medical school and residency training should prepare PCPs for appropriately managing common skin conditions, clinical variants may pose diagnostic challenges. An example of a rare variant of a relatively common condition is the patch form of GA. It may be confused for more severe and even detrimental diseases such as MF. Because history and physical exam alone may not be enough to establish the diagnosis of patch GA, histologic evidence via skin biopsies are often necessary.

Histologic evaluation using H&E staining of the patient’s rash in this report revealed granulomas and degraded collagen consistent with the diagnosis of patch GA. Initial therapy for this condition is often high potency topical corticosteroids, such as betamethasone dipropionate 0.05% ointment. UV phototherapy is also a common initial treatment, but it requires weekly on-site clinic visits, which may be difficult for patients who are caregivers for their loved ones, especially while in the setting of a global pandemic. While biopsies are not always necessary in establishing a diagnosis of a skin condition, PCPs should recognize their importance and when best to utilize them.

### Conflict of interest

None
